# The causal relationship between gut microbiota and bone mineral density: a Mendelian randomization study

**DOI:** 10.3389/fmicb.2023.1268935

**Published:** 2023-10-23

**Authors:** Ying Wang, Xuejian Zhang, Guangjun Tang, Pin Deng, Yuyan Qin, Jinglu Han, Shulong Wang, Xiaojie Sun, Dongxiao Li, Zhaojun Chen

**Affiliations:** ^1^Beijing University of Chinese Medicine, Beijing, China; ^2^Beijing University of Chinese Medicine Third Affiliated Hospital, Beijing, China; ^3^Institute of Basic Theory of Traditional Chinese Medicine, China Academy of Chinese Medical Sciences, Beijing, China

**Keywords:** gut microbiota, bone mineral density, Mendelian randomization, age, causal relationship

## Abstract

**Background:**

The gut microbiota has emerged as an intriguing and potentially influential factor in regulating bone health. However, the causal effect of the gut microbiota on bone mineral density (BMD) appears to differ throughout various life stages.

**Methods:**

We conducted a Mendelian randomization (MR) analysis to investigate the potential causal relationship between gut microbiota and BMD in five distinct age groups: 0–15, 15–30, 30–45, 45–60, and 60 years and older. The analysis employed three different methods, namely MR-Egger, weighted median, and Inverse-variance weighting, to ensure the robustness of our findings, a series of sensitivity analyses were also conducted, such as horizontal pleiotropy tests, heterogeneity tests, and leave-one-out sensitivity tests.

**Results:**

In the age group of 0–15 years, *Eubacterium_fissicatena*_group and *Eubacterium_hallii*_group were identified as risk factors for BMD. During the 15–30 age group, *Phascolarctobacterium*, *Roseburia*, and *Ruminococcaceae*_UCG_003 were found to be protective factors for BMD. In the 30–45 age group, *Lachnospira* genus demonstrated a protective effect on BMD, while *Barnesiella* and *Lactococcus* were identified as risk factors for BMD. Moving on to the 45–60 age group, *Eubacterium_ventriosum*_group, *Lachnospiraceae*_UCG_004, and *Subdoligranulum* were observed to be protective factors for BMD, while *Eubacterium_coprostanoligenes*_group, *Fusicatenibacter*, and *Lactococcus* were associated with an increased risk of BMD. In individuals aged 60 years and older, *Fusicatenibacter* and *Ruminococcaceae*_UCG_002 were also noted as risk factors for BMD. Conversely, *Eubacterium_ruminantium*_group, *Ruminococcus_gauvreauii*_group, *Alistipes*, and *Coprococcus*_3 were found to be protective factors for BMD, whereas *Barnesiella* and *Sellimonas* were identified as risk factors for BMD.

**Conclusion:**

A robust causal relationship between gut microbiota and bone mineral density (BMD) exists throughout all stages of life, with Firmicutes phylum being the primary group associated with BMD across age groups. Gut microbiota linked with BMD primarily belong to the Firmicutes phylum across age groups. The diversity of gut microbiota phyla associated with BMD depicts relatively stable patterns during the ages of 0–45 years. However, for individuals aged 45 years and above, there is an observed increase in the number of gut microbiota species linked with BMD, and by the age of 60 years, a trend toward an increase in the Bacteroidetes phylum categories is proposed.

## Introduction

The human skeleton plays a crucial role in maintaining overall health, with bone mineral density (BMD) serving as a major determinant of bone strength and fracture risk (Shevroja et al., 2021). The increasing prevalence of osteoporosis has garnered significant attention from the research community in recent years (Zhou et al., 2023). Throughout different life stages, BMD values vary, with rapid growth occurring during childhood and adolescence, culminating in peak BMD in early adulthood (Deng et al., 2021). However, as individuals age, hormone levels, such as estrogen and testosterone, decline, leading to a subsequent reduction in BMD (Ou et al., 2022). This loss of bone mass can increase the likelihood of developing conditions like periodontitis and arthritis while raising the risk of fractures (Hartley et al., 2022; Yu and Wang, 2022). Consequently, understanding the underlying factors impacting bone health and developing effective interventions to slow bone mineral loss is crucial for promoting healthy aging and reducing disease burden.

The gut microbiota, consisting of trillions of microorganisms including bacteria, fungi, and archaea, forms a symbiotic relationship with the human host and plays a vital role in maintaining overall health. The musculoskeletal system, particularly the skeleton, serves as a foundation for the human body. Recent research has highlighted a significant link between the gut microbiota and the skeleton, such as in the regulation of healing and remodeling, which can serve as fracture risk biomarkers (Hernandez, 2017). The potential role of the gut microbiota in osteoporosis, osteoarthritis, rheumatoid arthritis, and osteosarcoma, among other skeletal disorders, has been confirmed (Chen et al., 2022). The gut microbiota plays an important role in improving bone mineral density to combat bone tumors and joint diseases. By promoting the proliferation of intestinal and colonic cells, mediating the stability of the gut microbiota, and supporting mineral absorption in the intestines, supplementation with probiotics can enhance the mechanisms of healthy gut microbiota interactions, thereby increasing bone mineral density (Seely et al., 2021). Furthermore, the development of osteosarcoma can alter the gut microbiome, indicating that there is also an interaction between osteosarcoma and the gut microbiota (Le et al., 2023). The gut microbiota contributes to bone homeostasis through biochemical processes involving the immune, metabolism, and endocrine systems. The “gut-bone” axis, in which the gastrointestinal tract regulates bone health, is of critical importance in this context (D’Amelio and Sassi, 2018; Castaneda et al., 2020; Lu et al., 2021). The changes in intestinal microbiota in the human body are associated with aging, however, there is no research on how these changes affect BMD.

Mendelian randomization has become an increasingly popular method for inferring causal relationships in whole-genome association research data. This methodology overcomes biases originating from confounding and reverse causality by employing single nucleotide polymorphisms as instrumental variables, thereby allowing for accurate investigation of causal relationships between exposure factors and outcomes (Skrivankova et al., 2021). Mendelian randomization has proven to be a valuable tool in assessing the links between gut microbiota and disease (Xu et al., 2021; Li et al., 2022c). Using this methodology, the causal relationship between gut microbiota and BMD was investigated in the present study of individuals belonging to five distinct age groups, including 0–15, 15–30, 30–45, 45–60, and 60+ years. A two-sample Mendelian randomization approach was utilized to gain insights into potential preventive measures against osteoporosis in different age groups by regulating gut flora. Valuable information on managing osteoporosis through modulating the gut microbiota was offered by the results of this study. The potential causal relationship between gut flora and bone health was illuminated, and the research has significant implications for the development of novel interventions aimed at mitigating age-related bone loss.

## Materials and methods

### The MR study’s assumptions and design

**FIGURE 1:**
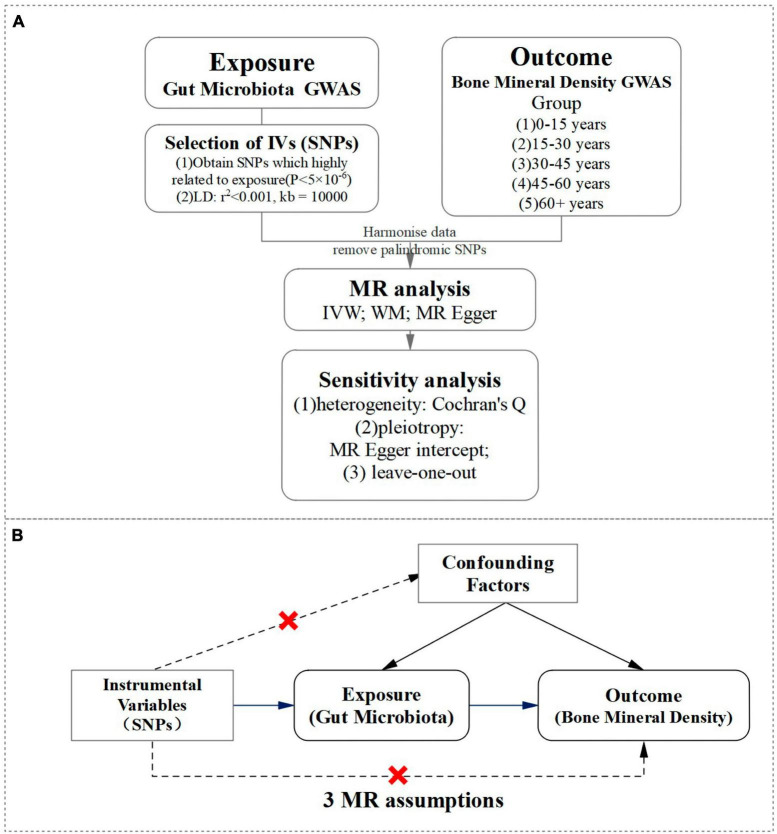
A visual representation of study design. **(A)** Flowchart of a two-sample Mendelian randomization design for gut microbiota and BMD. **(B)** The MR randomization analysis depended on three assumptions: (1) IV is significantly associated with gut microbiota. (2) IV is independent of confounding factors and unrelated to any confounding factors that affect the “exposure-outcome” relationship. (3) IV can only affect the outcome through exposure, and not through other pathways.

A two-sample Mendelian randomization approach was utilized in our study to investigate the potential causal association between gut microbiota and BMD across various age groups. Our objective was to explore the influence of gut microbiota on BMD at different stages of life. For a visual representation of our study design, please refer to Figure 1.

### Data sources

In this study, the gut microbiota data were sourced from the MiBioGen consortium, obtained through a large-scale ethnic GWAS analysis comprising 34,024 individuals from 18 cohorts (Kurilshikov et al., 2021). The BMD data were extracted from the IEU Open GWAS^1^ development database with single nucleotide polymorphisms (SNPs) as instrumental variables, where gut microbiota was the exposure factor, and BMD at five different age intervals (0–15, 15–30, 30–45, 45–60, and 60+ years) were the outcome factors.

### Instrumental variable selection

In our gut microbiota dataset, two hundred and eleven bacterial classifications ranging from phylum to genus were identified, however, unknown classifications were excluded, resulting in 117 bacterial genera being utilized in our MR analysis (Xu et al., 2021). We selected SNPs (IV) related to exposure from the gut microbiota GWAS dataset based on a *P*-value (*P* < 5.0 × 10^–6^), with a linkage disequilibrium (LD) coefficient of *r*^2^ < 0.001 and excluded the influence of LD on the results with a LD region length of 10,000 kb (Li et al., 2022c), to ensure independence between SNPs. Then, the SNP information related to both exposure was extracted and outcome and aligned the effect allele pairs for accurate dataset matching. It is crucial to adhere to the three assumptions of MR randomization analysis for the validity and reliability of this study. These assumptions are: (1) IV is significantly associated with gut microbiota. (2) IV is independent of confounding factors and unrelated to any confounding factors that affect the “exposure-outcome” relationship. (3) IV can only affect the outcome through exposure, and not through other pathways. Strict adherence to these assumptions allows for accurate conclusions and interpretations to be made.

### Statistical methods and sensitivity analysis

A comprehensive investigation was conducted into the potential causal relationship between gut microbiota and BMD using three different analytical methods: Inverse-variance weighted, MR-Egger, and weighted median. The primary method employed was the Inverse-variance weighted method (Xu et al., 2021). Heterogeneity in results was assessed using the *P*-value derived from the Cochran Q-test, where a *P* < 0.05 indicated the presence of heterogeneity, while a *P* > 0.05 represent no significant heterogeneity (Zhang et al., 2022). The reliability of MR analysis results was ensured by examining the intercept term of the MR-Egger method. A *P* > 0.05 for the intercept term indicated the absence of horizontal pleiotropy, which bolstered the robustness of the findings. The findings were authenticated by employing a leave-one-out methodology to progressively eliminate individual SNPs and ascertain if any anomalies had an impact on the results (Zhao H. et al., 2022). By observing the stability of the results after excluding these SNPs, the accuracy and consistency of the findings were ensured. All statistical analyses were conducted using R-4.2.3 and RStudio software, both featuring the Two Sample MR package. These rigorous methods and procedures aimed to enhance the scientific quality and credibility of the study on the potential causal relationship between gut microbiota and BMD.

## Results

### MR analysis results of the relationship between gut microbiota and BMD from ages 0 to 15

**FIGURE 2:**
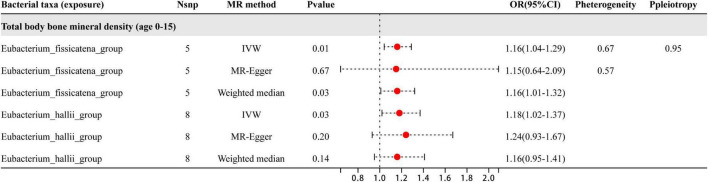
This plot displays the results of three different analyses (IVW, MR-Egger, and weighted median) investigating the relationship between gut microbiota and BMD in children aged 0–15 years. The red dot located to the right of 1.0 indicates that the gut microbe is a risk factor for BMD. The heterogeneity and pleiotropy are the results of sensitivity analysis.

The study analyzed a total of 20 genera, with results shown in Figure 2 revealing that only two specific genera of gut microbiota were found to have a causal relationship with BMD in the 0–15 year age group. The two genera were *Eubacterium_fissicatena*_group and *Eubacterium_hallii*_group, and the IVW estimates indicated that these genera were potentially harmful for BMD, acting as risk factors. Besides the sensitive analysis shows that the results do not have heterogeneity and pleiotropy.

### MR analysis results of the relationship between gut microbiota and BMD from ages 15 to 30

**FIGURE 3:**
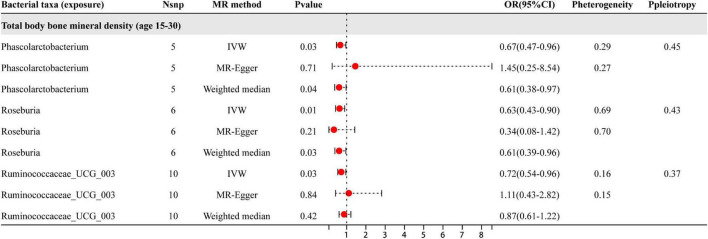
The results of three different analyses, namely IVW, MR-Egger, and weighted median. The red dots located on the right side of 1.0 indicates that gut microbiota is a risk factor for BMD, while those located on the left side represent protective effects (primarily observed in IVW results). The heterogeneity and pleiotropy are the results of sensitivity analysis.

Based on the results shown in Figure 3, three specific genera of gut microbiota were found to have a causal relationship with BMD in the 15–30 year age group. The IVW analysis indicated that the *Phascolarctobacterium*, *Roseburia*, and *Ruminococcaceae*_UCG_003 genera had suggestive protective effects on BMD. Besides the sensitive analysis shows that the results do not have heterogeneity and pleiotropy.

### MR analysis results of the relationship between gut microbiota and BMD from ages 30 to 45

**FIGURE 4:**
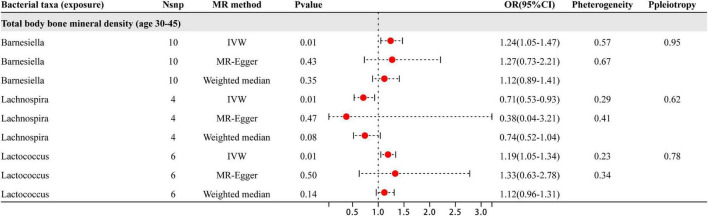
This illustration displays the findings from three distinct analyses: IVW, MR-Egger, and weighted median. The red dots positioned to the right of 1.0 indicate that gut microbiota is associated with an increased risk of BMD, while those on the left side represent protective effects (particularly observed in IVW results). Sensitivity analysis has revealed that the heterogeneity and pleiotropy observed are the outcomes of the analysis.

Based on the results shown in Figure 4, three specific genera of gut microbiota were found to have a causal relationship with BMD in the 30–45 year age group. The results from the IVW analysis indicated that the *Lachnospira* genus had a suggestive protective effect on BMD, while *Barnesiella* and *Lactococcus* were identified as risk factors for BMD. Besides the sensitive analysis shows that the results do not have heterogeneity and pleiotropy.

### MR analysis results of the relationship between gut microbiota and BMD from ages 45 to 60

**FIGURE 5:**
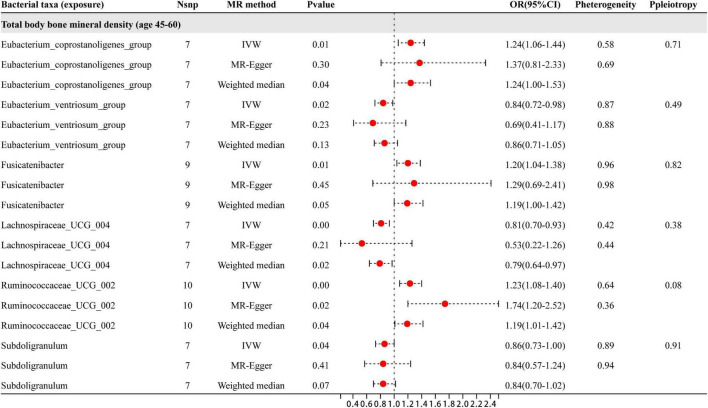
This figure presents the results of three different analyses, namely IVW, MR-Egger, and weighted median. The red dots located on the right side of 1.0 indicates that gut microbiota is a risk factor for BMD, while those located on the left side represent protective effects (primarily observed in IVW results). The heterogeneity and pleiotropy are the results of sensitivity analysis.

Based on the results shown in Figure 5, six specific genera of gut microbiota were found to have a causal relationship with BMD in the 45–60 year age group. The results from the IVW analysis indicated that the *Eubacterium_ventriosum*_group, *Lachnospiraceae*_UCG_004, and *Subdoligranulum* genera had a suggestive protective effect on BMD, while *Eubacterium_coprostanoligenes*_group, *Fusicatenibacter*, and *Ruminococcaceae*_UCG_002 were identified as risk factors for BMD. Besides the sensitive analysis shows that the results do not have heterogeneity and pleiotropy.

### MR analysis results of the relationship between gut microbiota and BMD in individuals aged 60 and above

**FIGURE 6:**
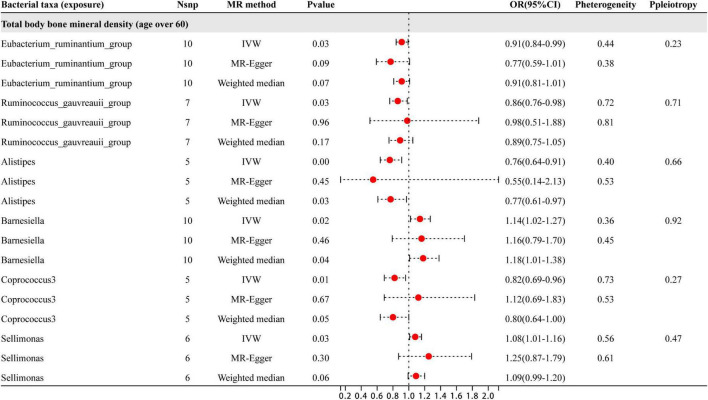
This diagram presents the results of three separate analyses: IVW, MR-Egger, and weighted median. The red dots located to the right of 1.0 signify that gut microbiota is linked to a higher risk of BMD, while those on the left side represent protective effect (particularly observed in IVW results). The heterogeneity and pleiotropy are outcomes derived from sensitivity analysis.

As shown in Figure 6, six specific genera of gut microbiota were found to have a causal relationship with BMD in people aged 60 years and older. The results from the IVW analysis indicated that the *Eubacterium_ruminantium*_group, *Ruminococcus_gauvreauii*_group, *Alistipes*, and *Coprococcus*_3 genera had a suggestive protective effect on BMD, while *Barnesiella* and *Sellimonas* were identified as risk factors for BMD. Furthermore, the sensitivity analysis showed that none of these results were influenced by heterogeneity or pleiotropic effects. Besides the sensitive analysis shows that the results do not have heterogeneity and pleiotropy.

**FIGURE 7:**
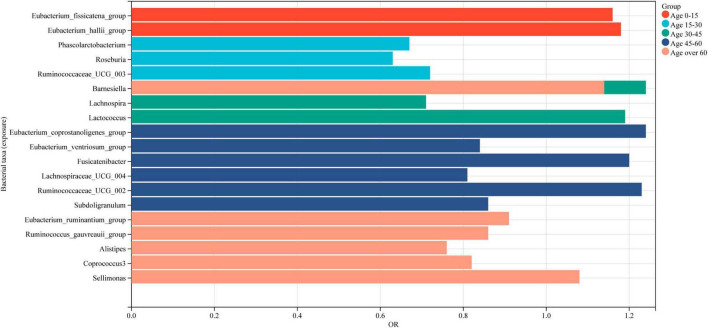
Intestinal microbiota associated with BMD in populations of different age groups. Red represents the gut microbiota associated with BMD in individuals aged 0–15 years, while light blue indicates those associated with BMD in individuals aged 15–30 years. Green signifies the gut microbiota associated with BMD in individuals aged 30–45 years, followed by dark blue for those aged 45–60 years, and finally, light red for individuals aged 60 years or older.

In this study, a two-sample Mendelian randomization analysis approach was utilized to investigate the potential causal link between BMD and gut microbiota. A total of 20 microbial genera were revealed to exhibit a significant causal association. The study findings represent that specific gut microflora genera may have a positive or negative impact on BMD for individuals of all age groups. Specifically, *Phascolarctobacterium*, *Roseburia*, *Ruminococcaceae*_UCG_003, *Lachnospira*, *Eubacterium_ventriosum*_group, *Lachnospiraceae*_UCG_004, *Subdoligranulum*, *Eubacterium_ruminantium*_group, *Ruminococcus_gauvreauii*_group, *Alistipes*, and *Coprococcus*_3 were identified as being protective for BMD, whereas *Eubacterium_fissicatena*_group, *Eubacterium_hallii*_group, *Barnesiella*, *Lactococcus*, *Eubacterium_coprostanoligenes*_group, *Sellimonas*, *Fusicatenibacter*, and *Ruminococcaceae*_UCG_002 were found to be risk factors (Figure 7).

It is notable that all the identified genera, except for *Barnesiella* and *Alistipes*, belong to the Firmicutes phylum. A healthy gut microbiota is typically dominated by the Firmicutes and *Bacteroides* phyla, with a small proportion of Actinomycetes and Aspergillus. It is important to note that the phylum-related diversity of the gut microbiota that is causally associated with BMD remains relatively stable between the ages of 0 and 45. However, over the age of 60, an increase in the number of *Bacteroides* species was observed in addition to the phylum Firmicutes genera that are causally associated with BMD. The new findings provide important insights into the complex link between gut microbiota and bone health, particularly in the elderly population. It offers a foundation for the development of targeted interventions for effective management of osteoporosis.

## Discussion

The composition of gut microbiota is influenced by various factors, including age, gender, medication, disease, and diet (Gioia et al., 2020). With advancing age, the intestinal barrier becomes more permeable, facilitating the passage of small molecules and bacteria through it. This increased permeability has the potential to induce abnormal immune reactions and subsequently lead to alterations in physiological functions (Thevaranjan et al., 2017; Konjevod et al., 2021). In early life, the diversity of gut microbiota is low (Whisner and Castillo, 2018), but over time, the gut microbiota gradually becomes more stable in adults (Zoetendal et al., 1998). However, during aging, gastric and intestinal degeneration and lesions may occur due to physiological decline, diet, and food types, leading to a reduction in the abundance of beneficial microorganisms in the gut microbiota of the elderly population.

The gut microbiota and bone have a vital connection that can act as a biomarker for fracture risk (Hernandez, 2017). The immune system and gut microbiota share an essential role in maintaining bone homeostasis (Pacifici, 2018; Seely et al., 2021). Certain strains of gut microbiota prompt the intestinal and systemic immune responses, thereby leading to the modification of remote organs and systems (Takiishi et al., 2017). The main metabolites produced by gut bacteria during dietary fiber fermentation are short-chain fatty acids (SCFAs) (den Besten et al., 2013). By stimulating the immune system, SCFAs entice immune cells to release anti-inflammatory molecules which alter the physiological characteristics of the bone (Schroeder and Bäckhed, 2016). Previous studies have suggested that SCFAs impact BMD by influencing host endocrine factors connected to bone metabolism (Chen et al., 2017). Experiments conducted on mice have indicated that regular antibiotic treatment can reduce IGF-1, resulting in reduced bone formation. As compared to mice treated without antibiotics, supplementation with SCFAs can restore IGF-1 levels and enhance bone mass (Yan et al., 2016).

Short-chain fatty acids (SCFAs), including acetate, propionate, and butyrate, have significant effects on gut health (Chambers et al., 2018). Butyrate, in particular, can promote the healing of intestinal inflammatory mucosa by stimulating the migration of intestinal epithelial cells (van Vliet et al., 2010). Firmicutes phylum is predominant in the production of butyrate, with genera such as *Lachnospiraceae*, *Roseburia*, *Eubacterium*, *Coprococcus*, and *Subdoligranulum* (Vital et al., 2014; Singh et al., 2023). Bacterial translocation is inhibited and intestinal dysbiosis-induced mastitis is reduced by *Roseburia* through the production of butyrate in mice (Zhao C. et al., 2022), while inflammation and immune responses in humans are regulated by *Eubacterium* through the modulation of SCFAs, cholesterol, and bile acid metabolism (Mukherjee et al., 2020). An experimental study showed that intervention with SCP-1 significantly enhanced the growth of *Eubacterium_ventriosum*_group in the intestine of Alzheimer’s disease rats, which facilitated the synthesis of butyrate (Zhang et al., 2023). Moreover, *Eubacterium_ventriosum*_group exhibited a negative correlation with inflammatory mediators IL-6 and IL-8 (Biagi et al., 2010), indicating that an increase in the abundance of *Eubacterium_ventriosum*_group can suppress inflammation. *Eubacterium_ruminantium*_group has the ability to produce short-chain fatty acids (SCFAs), which play a crucial role in energy balance, colonic motility, immune control, and inhibition of intestinal inflammation (Mukherjee et al., 2020). It can stimulate the development of colonic Treg cells and reduce inflammation (Smith et al., 2013). Animal experiments with VK2 in mice with colitis increased the abundance of *Eubacterium_ruminantium*_group in the colon, alleviating colitis by promoting dominant gut microbiota and regulating SCFAs, inflammatory factors, and intestinal barrier protein expression (Hu et al., 2023). Huang et al. (2020) suggested that *Ruminococcus_gauvreauii*_group’s relative abundance is positively correlated with the degree of host metabolic disorder. A positive correlation has been observed between the production of short-chain fatty acids (SCFAs) and *Ruminococcus gauvreauii* (Lippert et al., 2017). Overall, these findings represent that SCFAs and certain bacterial genera are essential for gut homeostasis and should be taken into consideration while addressing gut health. Researchers found through a randomized controlled trial that the probiotic group was able to promote an increase in the abundance of *Ruminococcaceae*_UCG_003, which can produce short-chain fatty acids (SCFAs) (Bloemendaal et al., 2021). In an animal study, a compound deer bone extract (CDBE) was found to improve postmenopausal osteoporosis symptoms by increasing the relative abundance of beneficial bacteria like *Alistipes* (Xue et al., 2021). This highlights *Alistipes*’s mediation role in osteoporosis. Another study confirms that *Phascolarctobacterium* can generate SCFAs like acetate and propionate (Wu et al., 2017). Inflammation is an important factor behind bone loss, but the aforementioned bacterial genera demonstrate protective effects on BMD through their ability to produce SCFAs, especially butyrate, by functioning as anti-inflammatory agents. This research provides evidence that specific gut bacteria producing SCFAs, particularly butyrate, have a protective effect on BMD. The anti-inflammatory properties of these SCFAs provide a potential therapeutic approach for the treatment of bone-related disorders. Further research should aim to elucidate the therapeutic potential of these bacterial genera and their SCFA products, including butyrate, in human subjects.

The gut microbiota plays a crucial role in bone healing and remodeling by affecting osteoclast activity and inhibiting osteoblast development. The relationship between gut microbiota and the skeleton was first established by Sjögren et al. (2012), who noticed that germ-free mice showed reduced numbers of CD4+ T cells in their bone marrow, lowered levels of TNF-α, and reduced expression of IL-6. This led to a decrease in osteoclast precursor cells and an increase in bone mass, signifying that the symbiotic gut microbiota stimulates bone resorption while inhibiting bone formation, ultimately reducing bone mass (Sjögren et al., 2012). The findings indicate that TNF-α can increase receptor activator of NF-κB ligand signaling, leading to bone loss and inhibition of mesenchymal stem cell differentiation into osteoblasts, causing a decrease in BMD and a disruption of the bone formation process (Kitaura et al., 2013; Gaffney-Stomberg, 2019; Mazziotta et al., 2022). *Eubacterium_hallii*_group serves as one of the butyrate producers of the infant gut and is essential for the symbiotic phenomenon that occurs during early life stages (Pham et al., 2016). The presence of *Ruminococcaceae*_UCG_002 in the gut influences the structure and function of the gut microbiota, ultimately leading to IgE-mediated food allergies (Lee et al., 2021). Rabdosia serra can alter the gut microbiota composition by increasing bacterial abundance and diversity, promoting beneficial bacteria, and decreasing pathogenic bacteria such as the *Eubacterium_fissicatena*_group, thereby supporting gut microbiota homeostasis (Li et al., 2022a). The available evidence suggests that bacteria from the Desulfovibrionaceae family and *Eubacterium_coprostanoligenes*_group contribute to producing trimethylamine/trimethylamine N-oxide, which is a risk marker for the development of atherosclerosis (AS) and cardiovascular disease (CVD) (Koeth et al., 2013; Rath et al., 2017). Conversely, high levels of *Eubacterium_coprostanoligenes*_group are linked with obesity, contributing to an AS risk factor (Gomes et al., 2018). A retrospective study has linked *Lactococcus* to endocarditis, hepatobiliary infections, and peritonitis, indicating its pathogenicity for various infections (Shimizu et al., 2019). In a clinical trial for ulcerative colitis, *Fusicatenibacter* abundance was found to be associated with the disease (Weng et al., 2019). *Sellimonas* has been shown to be related to inflammation and can increase in inflammatory diseases such as ankylosing spondylitis, atherosclerosis, and cirrhosis, especially after gut dysbiosis (Nayfach et al., 2019; Muñoz et al., 2020). *Barnesiella* belongs to the Porphyromonadaceae phylum and is a relatively abundant genus in the human gut microbiota, which has been linked to inflammatory bowel disease (Hahnke et al., 2016). Intraosseous inflammation can lead to abnormal bone remodeling and bone loss (Tilg et al., 2008). Reduction of inflammatory cytokines is important for maintaining bone health and reducing the risk of osteoporosis (Charatcharoenwitthaya et al., 2007). Similarly, a study by Di Stefano et al. (2001) found that excessive bacterial growth in the gut was associated with bone loss in the lumbar spine and femoral neck. Excessive bacterial overgrowth in the small intestine may occur in conditions of low BMD due to high levels of inflammation such as TNF-α and IL-1, promoting osteoclast activation (Stotzer et al., 2003). These findings represent that excessive growth of the gut microbiota may be a significant risk factor for osteopenia/osteoporosis. Therefore, efforts to decrease inflammatory cytokines are crucial for maintaining bone health and reducing the risk of osteoporosis.

The modulation of osteoporosis by probiotics has become increasingly popular in clinical practice. In a systematic review study (Huang et al., 2022), it was found that gut flora significantly improves bone development in infants, children, and adolescents, and that gut flora interventions serve as effective supplementary supplements for bone development. Furthermore, clinical trial studies have demonstrated that supplementation with probiotics enhances bone metabolism and increases the abundance of SCFAS-producing bacterial species, ultimately reducing bone loss. For instance, supplementation with Lactobacillus reuteri ATCC PTA 6475 and Bifidobacterium lactis Probio-M8 have demonstrated reduced bone loss in older women with low BMD and in menopausal women (Li et al., 2022b; Zhao et al., 2023). The long-term use of medications for osteoporosis treatment often leads to side effects. Therefore, targeting the gut microbiota may offer a potential alternative for treating these patients. In a randomized controlled trial, a combination of prebiotics/probiotics preparations with zoledronic acid and calcitriol showed a high response rate in treating primary osteoporosis patients by improving both bone metabolism and gut flora (Jia et al., 2022). This clinical evidence underscores the potential of the gut microbiota as a therapeutic target in the treatment of osteoporosis.

Our study has several limitations that warrant consideration. Firstly, the data utilized in this research were obtained from publicly available databases, and our analysis was based solely on statistical methods. While this strategy offers valuable insights, further experimental studies, particularly randomized controlled trials, are imperative for a more comprehensive understanding of the fundamental biological mechanisms at play. Secondly, incorporating multi-omics approaches, such as metagenomics, metatranscriptomics, and metabolomics, would be instrumental in unraveling the intricate interactions between the gut microbiota and BMD. By integrating these high-throughput techniques, we can attain a more holistic perspective on the functional roles of diverse microbial taxa and their contributions to host health or disease. Furthermore, it is pivotal to conduct a comprehensive exploration of the integrated effects of different taxonomic levels within the gut microbiota. This encompasses not only the genus level but also higher taxonomic ranks, including phylum, order, and family. Examining the impacts of these taxonomic levels will yield a more refined and nuanced comprehension of the relationship between gut microbiota composition and BMD. In summary, although our research imparts valuable insights into the gut microbiota, it is crucial to acknowledge these limitations and address them through further experimental studies, exploration of higher taxonomic levels, incorporation of multi-omics approaches, and consideration of the broader microbial diversity within the gut ecosystem.

## Conclusion

The research results show that the effect of gut microbiota on bone density is relatively stable in the age group of 0–45 years. However, with increasing age, the types of gut microbiota affecting bone density significantly increase in those aged 45 and above. This highlights the importance of paying attention to gastrointestinal health in this age group. Furthermore, among those aged 60 and above, there is a trend of increasing prevalence of Bacteroidetes phylum categories, which affect BMD. This group of people also experiences a decline in their basic metabolic rate and should reduce their intake of high-fat foods. Overall, this study provides valuable insights into the intricate relationship between gut microbiota and BMD, highlighting the potential of regulating gut flora for preventing osteoporosis.

## Data availability statement

Publicly available datasets were analyzed in this study. This data can be found here: https://gwas.mrcieu.ac.uk/.

## Author contributions

YW: Conceptualization, Data curation, Investigation, Methodology, Software, Supervision, Visualization, Writing – original draft, Writing – review and editing. XZ: Data curation, Methodology, Supervision, Visualization, Writing – original draft, Writing – review and editing. GT: Data curation, Methodology, Supervision, Visualization, Writing – original draft, Writing – review and editing. PD: Data curation, Formal analysis, Writing – review and editing. YQ: Data curation, Formal analysis, Writing – review and editing. JH: Supervision, Writing – review and editing. SW: Supervision, Writing – review and editing. XS: Supervision, Writing – review and editing. DL: Supervision, Writing – review and editing. ZC: Methodology, Project administration, Supervision, Writing – review and editing.

## Footnotes

1
https://gwas.mrcieu.ac.uk/
